# Static and Dynamic Ultrasound Imaging to Visualize the Bladder, Bladder Neck, Urethra, and Pelvic Floor in Children with Daytime Incontinence

**DOI:** 10.3389/fped.2017.00247

**Published:** 2017-11-21

**Authors:** Rogier Schroeder, Keetje de Mooij, Luitzen Groen, Pieter Dik, Caroline Kuijper, Aart Klijn, Tom de Jong

**Affiliations:** ^1^Paediatric Urology, Emma Children’s Hospital, Amsterdam, Netherlands; ^2^Paediatric Urology, Wilhelmina Children’s Hospital, Utrecht, Netherlands

**Keywords:** ultrasonography, perineum, urinary incontinence, urology, bladder, diagnostic imaging

## Introduction

Daytime urinary incontinence is a major health care problem occurring in 15 and 5% of 4-year-old and 9.5-year-old children, respectively (1, 2). Continence comes from an intrinsic interaction between anatomical and functional factors. Ascertaining the cause of incontinence remains a challenging task and is often derived from a complex of features. A few principal causes are urge in overactive bladders, voiding postponement, dysfunctional voiding, an underactive bladder, and stress incontinence. Mostly no structural, neurogenic, or other organic cause can be found in children with daytime urinary incontinence. Bladder neck competence is an important factor in achieving continence. The following basic outlet functions are known: closure during urine storage with increased closure during exercise or bladder filling (the guarding reflex), sustained opening when voiding, and transient opening for males when ejaculating (3, 4).

Standardization of terms as well as diagnostic and therapeutic abilities have been improved by the efforts done by dedicated institutions (5). Essentially non-invasive screening is standardized consisting of history-taking, clinical examination, urinalysis, uroflowmetry, ultrasound (US) (including post-void residual volume), voiding, and stool diary and pressure-flow studies (1, 6, 7). Initial management requires basic diagnostic tests. Treatment is mostly conservative and empiric (lifestyle interventions, physiotherapy, and pharmacotherapy). More elaborate assessment is required when primary therapy fails, diagnosis is unclear, or symptoms and signs are complex/severe (8). More (and novel) diagnostic tools to pinpoint the reason for incontinence need to be evaluated. After all, only the right diagnosis can lead to appropriate therapy or even surgery.

Ultrasound can be a valuable and patient friendly additive to physical examination. Already, it is the primary imaging tool in children with urological problems. Physical examination can be stressful especially for the pediatric population. US is well tolerated, not expensive, widely available, not invasive, and needs no radiation (9). Nowadays, a portable handheld pocket US machine is available. Transperineal US provides accurate visualization, both static and dynamic. It provides anatomical and even functional information. Imaging the bladder neck is superior to transabdominal US due to the bony pubic landmark as a reference point (10). Also it seems better than cystoscopy since it provides an overview of the bladder neck and surrounding tissue instead of the rather limited internal view. Furthermore, US requires no anesthesia, making the situation more physiological enabling dynamic testing.

This article focuses on US imaging of the lower urinary tract for patients with daytime incontinence. It provides practical guidance on how to perform transabdominal and transperineal US. We combine current insights from the literature with our clinical practice. We like to promote the use of US for physicians specialized in urinary incontinence.

## US of the Urinary Tract

Ultrasound is commonly the first way of imaging in pediatric urology. Resolution of US is reasonably high due to low fat percentage and short stature. Its outcome may lead to further imaging.

A few basics about US should be mentioned. First, probe gel is crucial for high-quality US, so use it sufficiently. Hygiene of probes should be taken into account, especially when used for genital parts. Probes may act as a medium for bacterial contamination and transmission. To reduce such risk, one should apply a plastic sheath or glove (easily available). A high level disinfection between two procedures is mandatory (11).

Evidently prior to genital examination, children should be informed appropriately, and parents need to give consent. For US of the urinary tract in children, a high-frequency probe is needed (7 MHz and higher) (12, 13). The US machine should be digital to enable image enlargement. Preferably the machine allows to record imaging to enable selection of images. With this “cine-loop” option, real dynamic imaging can be stored and reviewed digitally. Hereby, operator-dependent flaws can be obviated.

## Transabdominal

The transabdominal US shows the entire bladder and surrounding anatomy. For the lower urinary tract, the US probe is applied on the abdominal skin approximately 2 cm above the symphysis. Images can be performed in a sagittal and transversal plane.

Transabdominal US represents the gold standard procedure to measure both the bladder volume and the post-void residual urine (PVR). For proper imaging of the lower urinary tract, one should image the bladder in a full and an empty phase. In children, a PVR of more than 20 ml found on repetitive occasions indicates incomplete emptying.

Ureteroceles can be visualized preferably in the minimally filled low pressure bladder. Furthermore dilated (distal) ureters can be identified indicating either an efflux or a reflux problem now or in the past.

Color Doppler sonography can be a useful application, especially to rule out a ureter obstruction. It visualizes the inflow of urine (“jet”) into the urinary bladder. Doppler also differentiates between urinary tubes and blood vessels (14).

Bladder wall thickness is a much-debated issue. Infravesical obstruction may cause hypertrophy of the detrusor muscle. In the pediatric population, this is mostly caused by urethral valves, dysfunctional voiding, or detrusor sphincter dyssynergia (15). The determinant should be measured when the bladder is empty. Bladder wall thickness may be measured at any position. Therefore, the intraobserver variability is shown to be low. A threshold of 5 mm has been set to identify patients with infravesical obstruction (15, 16). Above 5 mm, a significant association with detrusor overactivity has been proven (17). Bladder wall hypertrophy has shown to be reversible after relief of the bladder outlet obstruction (18–20).

Finally, transabdominal US provides additional information on the bladder neck and possible constipation. The bladder neck can be visualized when rotating the probe toward the minor pelvis. It may be open (funnel shape) or closed. When it appears open, the patient can be asked to strain to close the bladder neck.

Measurements on the rectum are performed with a filled bladder at an angle of about 15° downward from the transverse plane. Visualization in girls is required just distal to the cervix, whereas in boys, the rectum is located dorsal to the trigonal fold. An impression of the bladder base and a rectal distension of more than 29–35 mm in the absence of the urge to pass stools is a strong signal for constipation as a comorbidity (21, 22).

## Transperineal

Only few centers utilize the transperineal US for static and dynamic assessment of the lower urinary tract (13). As opposed to transabdominal US, it focuses on the bladder neck rather than the entire bladder. Transperineal US is performed by placing the probe on the urethral meatus in girls or behind the scrotum in boys. Scanning is than carried out in the sagittal—mostly midsagittal—plane aligning the transducer with both the pubic synchondrosis and the posterior urethra. In boys, only little perineal pressure should be applied to avoid distortion of the urethral anatomy. Inverting the US images on the viewing provides a more anatomic presentation that corresponds to the usual orientation on voiding cystourethrograms (12).

Ultrasound is most ideally performed in semi-reclining position. This allows proper placement of the probe and enables patients to strain. After the age of 4, most children are willing to execute small assignments like coughing and straining on demand. The technique for transperineal US has been well described and illustrated in the literature (9, 13).

Several determinants can be visualized. At rest static features can be seen. Dynamic determinants emanate from variable conditions of the pelvic floor like stress, relaxation, or pressurizing moments.

A few determinants have been studied by our study group. Average urethral length in girls showed to be 23 mm at birth and 32 mm at 15 years of age. Therapy-resistant incontinence seems associated with a short urethral length (23). Furthermore, we showed that dedicated physical therapy is able to cure dysfunctional voiding by improving pelvic floor dysfunction (24).

An overview of both static and dynamic determinants of transperineal US is provided in Table 1.

**Table 1:** Static and dynamic anatomical determinants that may be visualized by perineal ultrasound.

Static determinants transperineal US	
**At rest**	
Urethra	– Length and width – Shape; straight or angled (e.g., anterior deviated urinary stream) – Sphincter volume – Pathological findings ○ Valves, polyp, syringocele, utriculus cyst, diverticula, stricture, insertion ectopic ureter or ureterocele, arteriovenous fistula or aneurysm, calculi, foreign body, distortion

Vagina	– Length and width – Cervix – Localization distal end vagina (urogenital sinus/cloaca) – Pathological findings ○ Hematocolpos, mucocele, foreign body, ectopic ureter ostium, tumor

Rectum	– Anterior wall (indication filling state) – Localization distal end rectal pouch (anal atresia)

Bladder neck	– Anatomic position; high/normal/low – Funnel (open bladder neck = V-aspect) – Vesicourethral angle; flat/steep (synonyms; urethrovesical, retrovesical, pubo-urethral, alpha angle)

**Dynamic determinants transperineal US**	

I Valsalva maneuver (pressurizing) and voiding (relaxation)	
II When straining, coughing and tapping on the abdominal wall	

Urethra	– Position; fixed vs hypermobile (for and backward motion, pubourethral distance) – Contraction and insertion of the bulbocavernosus muscles – Insertion puborectal muscle

Bladder neck/bladder dome	– Position; fixed vs (rotational) descensus (synonym; hyperdescensus, descent, cystocele, rotation, rotational angle, dynamic angle of urethral inclination, pubourethral angle) – Funneling; positive/negative (opening bladder neck) – Guarding reflex (increased closure at bladder filling) – Holding reflex (increased closure at pressure-increasing moments like coughing) – Detain manoeuver; positive or negative (pelvic floor contraction at will) – Sacral twitch/reflex (increased closure when tapping the abdominal wall) – Micturition reflex, sporadically seen in neonates (detrusor contraction and bladder neck opening)

## Hypothetical Case

It concerns an 11-year-old girl with persisting daytime incontinence despite in-hospital urotherapy training. She experiences stress incontinence that is worsening over the years. The girl is known to have generalized hyperlaxity. The pelvic floor physiotherapist has concluded that she has a normal pelvic floor function with maximal strength.

1. Transabdominal US:
  – Rectum diameter of 2 cm
  – Bladder volume of 120 ml
  – Slightly open bladder neck (inability to close at straining)
  – Bladder neck shows 3 cm descensus at Valsalva
2. Transperineal US:
  – Length urethra 28 mm
  – Flat vesicourethral angle
  – Distal insertion puborectal muscle
  – Funneling also when straining
  – Hypermobile urethra when coughing
  – Large cystocele at Valsalva
  – Little pelvic floor contractions, also a weak guarding and holding reflex

This concerns a 11-year-old girl with stress incontinence due to congenital hypermobile urethra with a cystocele.

Video urodynamics will be performed to rule out any further uropathology. Patient will be considered surgery.

## Discussion

Transperineal US is an imaging tool for static (anatomical) and dynamic (functional) evaluation of the bladder neck, urethra, and pelvic floor. It can extend your basic physical examination. Especially the pediatric population can benefit from it since it is minimally bothersome. US may reveal urethral hypermobility, (rotational) descensus, and funneling during pelvic floor muscle action (10, 25). Eventually, transperineal US may even eclipse invasive harmful investigations needing placement of catheters, radiation, or general anesthesia.

The majority of studies regarding transperineal US originates from the urogynecological literature. It focuses on stress incontinent women and their follow-up after surgery. In case of failure of urotherapy for incontinence pediatric guidelines indicate further investigation (e.g., video urodynamics, cystoscopy or lumbar spine MRI). Outcomes of transperineal US correlate well to urodynamics (18, 26). US may therefore become an alternative to urodynamics. Likewise, cystography (or retrograde urography) might be omitted since negative US findings are highly accurate in ruling out urethral anomalies in male (27).

At suspicion of an overactive bladder or stress incontinence, no standard imaging is obliged. These patients may benefit from transperineal US. Diagnoses of incontinence on urge may be supported by visualization of a bladder neck funnel. Likewise, stress incontinent patients may reveal descensus. Interestingly dynamic US can even help to differentiate between dysfunctional voiding and a neurogenic micturition disorder. Positive sacral reflex (arch S2–S4) testing on tapping the abdominal wall (twitch) indicates a neurogenic defect (28).

In our institute, transperineal imaging is predominantly employed in incontinent girls that are resistant to urotherapy. Gross uropathology has already been ruled out by standard US. Transperineal US can visualize determinants that may cause incontinence, like descensus or the inability to contract the pelvic floor on demand. Clinically, a stress test for urine loss (e.g., jumping up and down) can also be helpful for diagnosis. Although invasive, wearing a vaginal tampon can also help to diagnose the cause of incontinence.

Interobserver variability is always an issue in US. However, particularly transperineal US appears to have acceptable reproducibility of measurements. This is largely explained by the pubic bone that serves as a bony pelvic landmark (29, 30). More standardization and objectivation of parameters for the diagnosis of urinary incontinence is needed. For perineal US, this process has only just started. It will lead to more reliable and reproducible results. One of the parameters that already has been validated is the rectum diameter (21, 22). Furthermore, developments in computer-aided vector-based processing are ongoing. This should lead to quantified assessment of bladder neck dynamics. Studies reveal that this manner of processing is rather sensitive and specific (31, 32).

New US techniques for visualization of complex anatomy are successfully being introduced into pediatric urology (14). Contrast-enhanced US using agents like microbubbles is such an example (33). At the same time, exact relations between imaging outcomes and clinical symptoms remain unclear. To draw hard conclusions from just transperineal US is therefore still impossible (9).

In conclusion, this article comprises information on transperineal US from our experience employed in daily practice and from existing literature. Incontinence mostly ensues from a multiplicity of static (anatomical) and dynamic (functional) factors. Transperineal US is a promising imaging tool that is harmless, non-expensive, and broadly accessible. It can be an alternative to standard bothersome investigations and adds to basic physical examination. A process of standardization and objectivation of parameters should lead to quantification of measurements. As opposed to educated guessing, more accurate conclusion can then be drawn. Please start performing transperineal US, just do it.

## Author Contributions

Staff, contribution to theoretical background perineal ultrasound, co-writer, literature search.

## Conflict of Interest Statement

The authors declare that the research was conducted in the absence of any commercial or financial relationships that could be construed as a potential conflict of interest.
